# Emotional Regulation Interventions on Developmental Course for Preterm Children: A Systematic Review of Randomized Control Trials

**DOI:** 10.3390/children10030603

**Published:** 2023-03-22

**Authors:** Vincenza Dell’Aversana, Marco Tofani, Donatella Valente

**Affiliations:** 1 MSc in Rehabilitaiton Sciences for Healthcare Professions, Sapienza University of Rome, 00185 Rome, Italy; 2 Department of Human Neurosciences, Sapienza University of Rome, 00185 Rome, Italy

**Keywords:** preterm, children, emotional regulation, systematic review, rehabilitation, intervention

## Abstract

Children born preterm (<37 weeks of gestation) are at increased risk of socio-emotional difficulties. This study aims to determine the effects of rehabilitation intervention on the emotional regulation of children born preterm through a systematic review. We conducted a systematic review according to PRISMA guidelines. The literature screening was carried out on PUBMED, SCOPUS and WEB OF SCIENCE in August 2022. An author identified eligible studies based on predefined inclusion criteria and extracted the data. RCT quality was assessed using the JADAD and PEDro scales. We selected five RCTs for qualitative synthesis, having the common objective of evaluating the changes in emotional regulation after a rehabilitation intervention. Evidence of benefits was found after parent training intervention (PCIT; *p* < 0.05). Moreover, there was an improvement in day-to-day executive life and fewer behavioral problems after mindfulness intervention. Clinical, but not statistical, efficacy was found for the group-based physiotherapy intervention. In conclusion, parent training and mindfulness interventions can be helpful rehabilitation techniques, but the relatively small sample limited statistical power, so the discovery needs to be interpreted cautiously. Further research on these aspects is recommended.

## 1. Introduction

Children born preterm (<37 weeks’ gestation) show a specific vulnerability for socio-emotional difficulties, which may increase the likelihood of developing behavioral and psychiatric problems in adolescence and adulthood. The significant advances in perinatal and neonatal medicine over the past few decades mean that most of these infants now survive to adulthood [1,2,3]. Consequently, the focus of research has shifted from increasing survival rates to enhancing the quality of life and improving outcomes for these infants. It has been noticed that there is an increased risk of cognitive, behavioral, socio-emotional, speech, motor or sensory impairment in the long run [4,5,6,7]. Furthermore, long-term overall function depends on healthy socio-emotional functioning; at the same time, preterm children present more behavioral and emotional problems than their full-term counterparts [8,9,10]. The difficulties with the increasing requests, increasingly complex and demanding, will affect the learning, self-esteem and social development of the child and future adolescent [11].

This leads to emotion regulation, which refers to the ability to modulate emotions in response to people and situations, delay gratification and tolerate changes in the environment using behavioral processes and strategies and enabling appropriate empathic behaviors [12,13]. Then, emotion dysregulation can lead premature children to be unable to handle environmental stimuli, showing hyperactive responses and low tolerance to the slightest stimulation, putting children born prematurely at a disadvantage during social situations [14,15,16]. It has been found that several risk factors such as atypical structural maturation of the brain regions underlying social cognition could lead to developmental delays or abnormalities [17,18,19]. At age 6, reduced connectivity was found in all emotions except the response to angry faces. However, this issue with reduced connectivity decreases at 8 years, indicating a dynamic period of brain network development. The affected areas are the amygdala and the frontal regions, in particular the superior frontal gyrus and between the orbital frontal cortex and the anterior cingulate [20,21,22,23,24,25]. Other volumetric alterations have been found in samples of preterm infants in infancy and adolescence with changes in both white and gray matter [17]: reduced volume of the fusiform gyrus [26,27], thalamus [19], insula [26] and hippocampus [28,29,30,31]. However, different studies have identified, in addition to emotional dysregulation, an impairment of social cognitive skills related to the Theory of Mind [12,32]. Theory of Mind has been defined as the ability to understand the behavior, the inner state that guided it and the motivations and emotions of others, even when different from one’s own [32]. These impairments increase social vulnerability leading to the result of specific alterations of the “Social Brain”, which is considered as a neurodevelopmental sequela of preterm birth [33]. The Theory of Mind deficit is reflected in a reduction in the connectivity of a set of brain regions that comprise the “Social Brain”. These networks include regions such as the angular gyrus, medial prefrontal cortex, superior temporal gyrus and temporal lobes. This deficit is particularly found in the group of children born very preterm, who showed greater connectivity than controls in a network anchored in the occipital gyrus rather than in the classic regions of social processing [34].

It is known that behavioral problems are frequent as early as 2 years of age and that these deficits found in the preschool age remain stable in early childhood and persist throughout the school, adolescent and adult years [35,36,37,38]. This could evolve into psychiatric disorders, particularly anxiety disorders, which are the most prevalent disorder in this population [39,40]. In fact, during the first 2 years of life, higher rates of insecure attachment with parents were recorded compared to term children and greater difficulties in regulating interactions with primary caregivers [41]. In the following years, on the other hand, greater problems in behavioral and emotional self-regulation, less empathy and lower levels of motivation for the task, social interactions and prosocial relationships between peers were highlighted, also confirmed by the evaluations of parents and teachers. This is particularly true for children born extremely preterm in preschool and school age [12,15,38,42]. Arriving at adolescence, we find a developmental period characterized by an increase in cognitive and emotional self-regulation, a greater dependence on peers for socialization and a greater sensitivity to reward and socio-affective stimuli. The neural network continues to develop during this time and supports greater awareness of mental states and intentions. Despite the aspects that promote the growth of adolescents’ increased sensitivity to reward and affective stimuli, these changes increase vulnerability to stress and the possibility of making decisions with negative adaptive consequences [15,16]. Nevertheless, it should be noted that regulatory processes vary during development. In addition to time, the effects on regulatory functions of environmental quality, modification of parental interactions and experiences can improve the balance between biological and environmental regulation by shifting in the direction of environmental dispositions, thus modifying developmental outcomes [12,43]. In this way, psychosocial variables such as positive parenting interactions or low parental stress that can potentially protect preterm children from behavioral problems acquire considerable importance [11,44,45]. Furthermore, it is believed that interventions should also aim at recognizing emotions and elaborating on the emotions felt by others [22]. A comprehensive approach inspired by a bio-psychosocial model of health and the International Classification on Functioning Disability and Health is urgently recommended [46]. Researchers suggest that the behavioral phenotype is characterized by inattention, anxiety and social difficulties, and that these characteristics would remain stable in early childhood and persist throughout school age, adolescence and adulthood [37]. In particular, emotion dysregulation can lead these children to be unable to manage environmental stimuli, showing hyperactive responses and low tolerance to minimal stimulation [14].

Pediatric rehabilitation is a discipline that enables children with acquired or congenital disabilities to reach their maximum physical, mental, social, occupational and educational potential. It is a specialized field that requires adequately equipped healthcare workers [47,48]. Instead, psychoeducation is defined as an intervention that integrates emotional and motivational aspects to enable patients to cope with the illness or their difficulties and to improve treatment adherence and efficacy [49]. For these reasons, this systematic review aims to identify the available rehabilitative and psychoeducational interventions that modify emotional regulation in preterm children, evaluating the potential benefits they can offer in this specific population. 

## 2. Materials and Methods

The systematic review was performed following the PRISMA (Preferred Reporting Items for Systematic Reviews and Meta-Analyses) statement [50]. The research group has experience performing systematic reviews of developmental age and validating specific outcome measures [51,52,53,54,55,56].

### 2.1. Search Strategy and Eligibility Criteria

Electronic databases searched in August 2022 were MEDLINE (PubMed), Scopus and Web of Sciences. The following electronic search strategies were used: “Infant, Premature” [Mesh] AND “Emotions” [Mesh] AND (“Rehabilitation” [Mesh] OR “therapy” [Subheading]) and (“Emotional Regulation” [Mesh]) AND “Infant, Premature” [Mesh]. The search was conducted manually and adapted to the different databases as needed. Randomized controlled trials were included. Group comparisons, pre–post comparison designs, book sections and single-case study designs evidence were excluded. There were no filters or restrictions on language, the country where the study was conducted or the year of publication to avoid the loss of potentially embeddable documents. The population was limited to children born prematurely and aged over 3 years. We included all types of rehabilitation and educational interventions available and compared them with standard medical care, waiting lists and any other therapies or protocols carried out; outcomes included emotional and behavioral regulation improvements. Secondly, all the further functional modifications evaluated instrumentally and clinically were considered.

### 2.2. Study Selection and Data Collection Process

The database searching was performed manually, and the duplicates were manually excluded with Excel by an independent analysis performed by a reviewer. A single reviewer performed the study eligibility assessment and data extraction process. Following the guidelines of the PRISMA checklist [50], the first selection of studies was initially conducted considering the title, keywords and abstract, selecting the relevant documents according to the previously described inclusion and exclusion criteria; subsequently, the full-text articles were independently reviewed and included in the final list of eligible studies. In case of doubt of the first reviewer, the opinion of the second reviewer was used to achieve certainty. The following relevant characteristics of the included studies were then extracted: name of the first author and year of publication, participants, rehabilitation interventions of the experimental and control groups, duration of treatment and follow-up, outcome measures, and results (Table 1).

### 2.3. Risk of Bias

To evaluate the quality of the studies, the Jadad [62] and Physiotherapy Evidence Database (PEDro) scales were used, calculating the scores for each item included. The PEDro and Jadad scores are specifically designed to assess study quality and include specific questions to identify potential methodological biases. The Jadad score includes three elements: randomization, blinding and description of withdrawals and dropouts. One point is assigned for each item if an accurate and detailed description is provided, but this point is revoked if the item is judged inadequate or incomplete. The highest possible score is five points, and studies scoring less than three points are generally considered to be of low methodological quality. On the other hand, the “PEDro rating scale” comprises 11 criteria, including randomization, blinding of treatment participants, therapists or intention to treat analysis. The rating scale is a checklist of “yes or no” responses to each criterion, and the “yes” responses are added together. The highest score is 10, as the first item is not counted, and a score greater than 7 or equal to 7 is considered of high methodological quality [63]. In this review, the first reviewer assessed the risk of bias in the included studies and any concerns were resolved in consultation with the second reviewer.

## 3. Results

**Figure 1:**
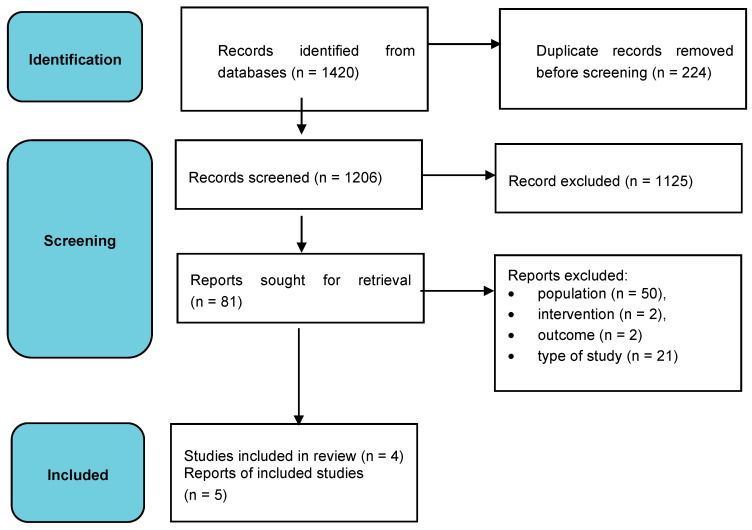
Prisma flow diagram.

The research identified a total of 1420 records corresponding to the selection criteria applied through database searches (Figure 1). The 214 duplicates were manually excluded, the remaining 1206 records were screened and a further 1125 of these were excluded due to the different populations not falling within the inclusion criteria, the type of intervention, the outcome analyzed or the type of study performed. The full texts of the five remaining studies [57,58,59,60,61] were included and assessed for qualitative synthesis.

The articles included in the qualitative analysis correspond to the applied inclusion criteria. All five articles are RCTs with patients born prematurely and over 3 years of age who have followed a rehabilitation or psychoeducational intervention and evaluated the changes at the level of emotional regulation. The studies were published between 2010 and 2020. Table 1 presents a data extraction of the included studies. In particular, the following data are reported: first author and year of publication, participants, rehabilitation interventions, duration of treatment and follow-up, outcome measures, and results. 

The sample size ranges from a minimum of 28 [59,60] to a maximum of 85 children born preterm [58], with age ranging from 3 [59,60] to 12 years [58,61]. Four of the five studies reported the duration of follow-up, which ranged from 1 to 12 months [57,58,60,61]. One study reported no follow-up [59]. The main objective of the three included studies was to evaluate emotional and behavioral changes in preterm patients following the interventions performed [57,59,60]; Siffredi (2020) also wanted to evaluate executive functions and Van Houdt (2019) also the attentional functioning and self-perception. Except for two studies including parents, the studies carried out different rehabilitation interventions aimed directly at the child or adolescent.

### 3.1. Group Physiotherapy Intervention

The RCT study performed by Brown et al. (2017) investigated behavioral and emotional regulation changes that task-oriented group physiotherapy intervention, combined with a home-based program, can determine over 6 weeks. Specifically, the task-oriented approach emphasizes motor performance and incorporates it into cognitive and attentional processes. Included activities addressed postural control and balance, sensorimotor skills and upper girdle strength, as well as behavior such as increasing attention to tasks. Fifty four-year-old children born extremely premature were recruited and randomized into the experimental (n = 24) and control (n = 26) groups. The latter received standard treatment through Best Practice advice and an informal booklet of general age-appropriate activities. There were no significant differences between groups over time on CBCL internalizing, externalizing or total problems scores. The intervention group showed a mean difference in total problems score of −3.8 (CI [1.5, 9.1]) between times, with standard care group values being −4.4 (CI [1.6, 7.1]). Males had higher total problems scores than females (*p* = 0.026), although still performed within the “normal” range. At the end of the treatment and the follow-up, carried out after one year, the authors recorded an improvement in both groups at the behavioral level, but no significant differences were identified between the two groups at the behavioral level of internalizing (*p* = 0.621), externalizing (*p* = 0.804) and problematic (*p* = 0.596). 

### 3.2. Computerized Intervention in Executive Functions

Van Houdt and colleague (2019) studied the effects of a computer-based intervention focused on executive functions (EF) in eighty-five children born very preterm between the ages of eight and twelve. Twenty-nine children were assigned to the experimental group and twenty-six to the placebo group; the remaining thirty were assigned to the waiting group. The experimental intervention and placebo involved a 6-week intervention, with 25 sessions ranging from 30 to 45 min. The experimental intervention applied the BrainGame Brain Training, a highly motivated computerized intervention that can be carried out independently by the child and which consists of exercises focused on executive functions. In the working memory task, children are asked to repeat a sequence of dots on a grid. In the inhibition task, children are asked to press a button in a specific time window (target), but to refrain from pressing that button when a visual stop signal is presented. In the cognitive flexibility task, children are asked to sort objects according to either its shape or its color, with the sorting rule changing every three to five trials. The difficulty level of each training task is automatically adjusted to the child’s level of performance. The placebo group carried out the same activities without reinforcing cognitive skills; furthermore, the difficulty level was non-adaptive, constantly remaining low. The authors found that the intervention group improved in performing all tasks (*p* < 0.001); however, these skills were not generalized across attention (*p* = 0.25), parent (*p* = 0.19)- or teacher (*p* = 0.62)-assessed behavioral and emotional functioning, or self-perceived competence (*p* = 0.12).

### 3.3. Parent Training

Two articles reported the results obtained from an RCT of parent training [59,60] involving twenty-eight children born preterm with an externalizing behavior disorder and their mothers. Fourteen mother–child pairs were assigned to the experimental group and the other fourteen were on the waiting list. The parent–child interaction therapy (PCIT) is a parent training intervention focused on enhancing the interaction of the mother–infant dyad; it consists of one session per week for a total of four months. Treatment progresses through two distinct phases: child-directed interaction (CDI) resembles traditional play therapy and parent-directed interaction (PDI) resembles clinical behavior therapy. Bagner et al. (2010) report that the PCIT group had fewer attention problems (*p* = 0.11), but most of all less aggressive behaviors (*p* < 0.05) and externalizing and internalizing behavior problems (*p* < 0.05) at the end of the sessions (F = 24.2, *p* < 0.05). The study by Rodriguez et al. (2014) reported how the PCIT group increased global regulation (*p* < 0.05); in particular, the resulting t-tests indicated that both low and high global regulation was significantly different from zero, t(26) = −7.38, *p* < 0.01, b = −100.72, and t(26) = −3.51, *p* < 0.01, b = −48.71.

### 3.4. Mindfulness Intervention

The cross-over RCT performed by Siffredi et al. (2020) investigates the effects of a mindfulness intervention on the emotional regulation of 56 adolescents born very preterm. The experimental group followed a mindfulness-based intervention (MBI) (n = 29) and was compared to a waitlist group (n = 27). The MBI intervention consisted of eight weekly sessions in groups of up to seven participants, lasting ninety min, plus an indication to practice daily at home. For each session, one theme was addressed, such as attention and the stabilization of the focus of attention, bodily sensations, emotions, thoughts, stress and coping strategies. Different formal meditation practices were introduced. Groups were evaluated at the end of the intervention and a follow-up at one and three months. The authors concluded that mindfulness improved day-to-day executive life and reduced SDQ scores (t = −2.423, *p* = 0.017); however, these improvements were not globally maintained at follow-up, except for information processing (t = −3.341, *p* = 0.001).

### 3.5. Methodological Quality and Risk of Bias

The methodological quality of the selected studies was assessed by applying the Jadad and PEDro scores to each of them. Three of the studies included in the present review, Brown et al. (2017), Siffredi et al. (2020) and Rodriguez et al. (2014), were considered low quality as they achieved a Jadad score of two and exceeded the PEDro scale cut-off. The remaining RCTs scored equal to or greater than the cut-off values and were rated as good quality (see Table 1). The main problems with the articles receiving low scores were the impossibility of applying a double-blind study due to the nature of the treatment, the inadequate description of the drop-out and the withdrawals. Quality assessments were initially completed by a single reviewer and then verified for accuracy by the second reviewer.

## 4. Discussion

The scientific literature has found that children born preterm are vulnerable to social–emotional difficulties, leading to an increased likelihood of developing behavioral and psychiatric problems in adolescence and adulthood [11,41]. Among the interventions available to limit and compensate for this vulnerability, the effectiveness of preventive interventions in NICU has been confirmed, which concerns the care of the relationship between parents and preterm infants [64,65]. Nevertheless, there are no indications regarding older children and the present systematic review seems to be the first in the literature to examine, through an analysis of RCTs, the efficacy of rehabilitative and educational interventions in improving emotional regulation in children born preterm over 3 years. The studies selected were very heterogeneous, making it impossible to compare the protocols and, consequently, the results achieved at a quantitative level. Instead, the results collected at a qualitative level showed insufficient evidence, and the main conclusions obtained from the five randomized controlled trials will be demonstrated.

The first selected RCT [57] investigated changes in behavioral and emotional regulation of a group physiotherapy intervention. Despite an improvement in behavior, no significant differences were identified compared to the control group. This may be related to the small sample recruited, which did not show emerging emotional regulation difficulties in the initial assessment. Regarding the impact of the intervention, the children benefited from the group physiotherapy and the guidance with which they were provided. However, the interpretation must be made with extreme caution given the low quality of the study performed.

The study that investigated the effects of a computerized intervention on executive functions was also ineffective [58]. The authors found that the intervention group improved in performing all tasks, but these abilities could not be generalized to the sample’s attention or behavioral and emotional functioning. This is also in line with what emerged from the recent meta-analysis by Sala et al. (2019), who state that executive function training programs produce reliable improvements that are difficult to generalize. Furthermore, there may be limits to the plasticity of the preterm infant brain that may influence how EF training improves the multiple functions investigated [66]. However, specific training programs based on executive functions and emotion recognition were developed for other populations and could be used as a baseline intervention [67,68].

Parent training was a third rehabilitation intervention investigated by two articles [59,60]. They found better regulation and fewer attention problems, aggressive behaviors, and externalizing and internalizing behavior problems that were maintained even after the intervention was carried out, thanks to positive interactions in the mother–child dyad that persisted over time. Children with poorer regulation have been found to benefit more from treatment. This can be related to the greater room for improvement and the high maternal motivation in implementing the proposed skills. In support of this, it has also been shown that the quality of parent–child interactions predicts the emerging ability to regulate emotions in children born prematurely and that they are particularly susceptible to the effects of negative early parenting [12,69]. Although a biological vulnerability is present, emotional dysregulation can also be influenced by some difficulties in parenting management. In fact, the results of the study carried out by Clark and colleagues (2008) showed that parents of extremely preterm infants had greater difficulty in modifying the interactions around their children’s cues and also in providing adequate timely support. Rather, they tended to become intrusive when solving their problems [12].

There is also strong empirical support for family-focused interventions for children with emotional, behavioral and relationship problems [70,71,72]. Nonetheless, the data must be interpreted with caution due to the small sample size and the reduced quality of one of the two selected studies.

The latest RCT [61] investigated the effects of a mindfulness intervention on emotional regulation. They showed short-term improvements, including general behavior, but they were not maintained over time except for the increase in processing speed. The initial encouraging results align with previous data in the literature showing improvement in behavioral skills in the short term after mindfulness intervention in full-term adolescents [73,74,75]. However, no significant effect on the quality of life and social skills was observed.

In the case of a child’s emotional regulation difficulty, it would be useful to work on family dynamics by focusing on the interaction between children and parents. Parents should acquire better communication skills to reflect the child’s claims and describe his or her behavior. Then, they should focus on learning to use effective commands and to constantly respect the timeout for non-compliance and negative behavior [60]. When the difficulties concern an adolescent, on the other hand, it is possible to refer to the concepts of mindfulness such as openness, non-judgment and acceptance by integrating these mechanisms with cognitive, emotional and self-related processes [76,77]. They initially focus on the present moment; then, they develop greater attentional and behavioral self-regulation and finally engage the participant in a sustained mindfulness meditation practice [78].

### Limitations of the Study

There are several limitations in this systematic review. First, the small number of randomized clinical trials found in the literature, which also had low participant numbers and high heterogeneity. The literature has mainly focused on the neonatal period and the interventions performed in the NICU together with the parents. For this reason, studies relating to older children are few, albeit increasing. Therefore, there is no possibility of having solid evidence. A second limitation relates to the design of the study, as some records were excluded because they were research protocols and characterized by the absence of includable results [79,80]. However, this also signals the emerging interest of the scientific community in interventions that can be implemented following the discharge of premature infants from the NICU. It is expected that more specific studies will emerge on this topic and be of greater methodological quality in the future. Another limitation of this work is that, although some studies have investigated the same outcomes, they have been measured with different instruments, making it impossible to compare and reach solid conclusions. Finally, not all databases have been included in this search strategy.

## 5. Conclusions

In conclusion, this systematic review indicates that the scientific community still needs to investigate the benefits that rehabilitative and educational interventions can bring in the emotional regulation of preterm children. Further studies should investigate these aspects in more depth. Among the available studies, those that address the entire family nucleus, such as parent training interventions, or which work on achieving greater emotional awareness, such as mindfulness, have proven to be possible approaches for enhancing emotional regulation because they have a more significant impact on the entire cognitive and affective–relational development. The promising results indicate that rehabilitation interventions may specifically strengthen protective factors, such as resistance to parenting stress or increased awareness, and reduce risk factors for social–emotional difficulties, such as negative parenting. However, these data must be taken with caution, given the heterogeneity of the studies and the small sample available, which make the evidence insufficient.

## Figures and Tables

**Table 1:** Data Extraction of Selected Studies.

Study	Sample	Intervention Group	Control Group	Duration and Follow-up	Outcome Measures	Results	Jadad	PEDro
Brown et al. (2017) [57]	50 4-year-old children ELBW IG:24; CG: 26	Group-based physiotherapy intervention	Standard care: best practice advice	6 group-based physiotherapy weekly sessions and home program F: 12 months	Child Behavior Checklist (CBCL); Movement Assessment Battery for Children Second Edition, Beery Visual-Motor Integration Test 5th Edition and Peabody Picture Vocabulary Test 4th Edition	IG and CG improved on CBCL total problems score at baseline and 1-year follow-up, *p* = 0.004. There were no significant differences between groups for CBCL internalizing, externalizing or total problems scores	2	6
Van Houdt et al. (2019) [58]	85 8–12-year-old children born very preterm IG: 29 CG: 26 (placebo training); 30 (waitlist)	EF training	Placebo training or waitlist	The EF and placebo training involved 6 weeks, for a total of 25 (30–45 min) sessions of the training program. F: 5 months	Child version of the Attention Network Test (Child-ANT), Strengths and Difficulties Questionnaire (SDQ) and Self-Perception Profile for Children (CBSK)	IG improved on all training tasks but not on attention, parent- or teacher-rated behavioral and emotional functioning, or self-perceived competence	5	9
Rodríguez et al. (2014) [59]	28 children born preterm with externalizing behavior problems and their mothers IG: 14 CG: 14	Parent training (PCIT)	Waitlist	One session a week for four months	Eyberg Child Behavior Inventory (ECBI) and videotaped 10 min parent–child interaction	PCIT increased global regulation (*p* < 0.05)	2	4
Bagner et al. (2010) [60]	28 children born preterm with externalizing behavior problems and their mothers IG: 14 CG: 14	Parent training (PCIT)	Waitlist	One session a week for four months F: 4 months	Eyberg Child Behavior Inventory (ECBI) and videotaped 10 min parent–child interaction	IG had fewer attention problems, aggressive behaviors, and externalizing and internalizing behavior problems	3	8
Siffredi et al. (2020) [61]	56 very preterm young adolescents, IG: 29, CG: 27	Mindfulness-based intervention (MBI)	Waitlist	8-week MBI in a cross-over design. F: 1 month F2: 3 months	Behavior Rating Inventory of Executive Function—parent version (BRIEF), Strength and Difficulties Questionnaire—parent version (SDQ), KIDSCREEN-27, NEPSY-II	IG enhanced organizational capabilities in everyday life and decreased SDQ scores, but it was not maintained at follow-up except for the improvement of information processing	2	6

ELBW: Extremely Low Birth Weight; F: follow up; CBCL: Child Behavior Check List; EF: Executive Function; PCIT: Parent–child Interaction Therapy; SDQ: Strength and Difficulties Questionnaire; NEPSY: NEuroPSYchological Assessment—2nd edition.
